# Intersectional Disparities in Digital Health and Mental Health Service Use Among US Youth During the COVID-19 Pandemic: Cross-Sectional Analysis of a National Survey

**DOI:** 10.2196/77062

**Published:** 2025-10-27

**Authors:** Meghan Romanelli, Carolina Vélez-Grau, Julien Rouvere, Sarah F Porter

**Affiliations:** 1 School of Social Work University of Washington Seattle, WA United States; 2 School of Social Work Boston College Chestnut Hill, MA United States; 3 Department of Psychiatry and Behavioral Sciences University of Washington School of Medicine Seattle, WA United States

**Keywords:** sexual minority youth, digital mental health services, digital health services, COVID-19, digital inclusion

## Abstract

**Background:**

Sexual minority youth, particularly sexual minority youth of color, report elevated mental health challenges and persistent barriers to care. The COVID-19 pandemic exacerbated these disparities and catalyzed a shift toward digital health and digital mental health services. This rapid transition has made it challenging to understand digital exclusion and the digital divide.

**Objective:**

This cross-sectional study identified the prevalence of digital health and digital mental health service use among US adolescents during the COVID-19 pandemic and examined heterogeneity by sexual orientation, race and ethnicity, and their intersection.

**Methods:**

Nationally representative data were obtained from the 2021 Adolescent Behaviors and Experiences Survey (N=7705). Weighted distributions of digital health and digital mental health use were calculated, and modified Poisson regression models estimated adjusted prevalence ratios (aPRs) by sexual orientation, race and ethnicity, and their intersection.

**Results:**

Across the sample, digital health and digital mental health use were 25.8% and 8.5%, respectively. (All percentages reported are weighted estimates.) Digital mental health use was 5.6% among heterosexual participants and 18.1% among all sexual minority youth. In adjusted models, sexual minority subgroups had higher prevalence of digital mental health use than heterosexual peers (lesbian, gay, and bisexual [LGB]: aPR 2.60; sexually diverse: aPR 2.41; all *P*≤.05). This pattern held among White, Black or African American, and multiracial LGB participants. Digital mental health use was 10.2% among White participants and ranged from 4.8% to 15% among racially or ethnically minoritized participants. Black or African American, Hispanic or Latino, and Asian or Pacific Islander participants had lower prevalence of digital mental health use than White peers overall (Black or African American: aPR 0.70; Hispanic or Latino: aPR 0.55; Asian or Pacific Islander: aPR 0.48; all *P*≤.05) and among sexual minority youth (Black or African American: aPR 0.60; Hispanic or Latino: aPR 0.35; Asian or Pacific Islander: aPR 0.23; all *P*≤.05). Racial and ethnic disparities in digital mental health use were pronounced among LGB (Hispanic or Latino: aPR 0.52; *P*≤.05) and sexually diverse participants (Black or African American: aPR 0.36; Hispanic or Latino: aPR 0.17; Asian or Pacific Islander: aPR 0.10; all *P*≤.05), but not heterosexual participants. Digital health use did not differ by sexual orientation. However, Black or African American and Hispanic or Latino participants had lower prevalence of digital health use than White peers (28.8%) overall (Black or African American: aPR 0.76; Hispanic or Latino: aPR 0.78; all *P*≤.05) and among heterosexual (Black or African American: aPR 0.73; Hispanic or Latino: aPR 0.80; all *P*≤.05) and sexual minority youth participants (Hispanic or Latino: aPR 0.75).

**Conclusions:**

Digital platforms offer promise for expanding access to mental health care among sexual minority youth, but persistent inequities must be addressed. Cocreation with lived-experience experts may be critical to ensure digital services are trusted, inclusive, and accessible for all youth.

## Introduction

### Background

Sexual minority youth, including lesbian, gay, and bisexual (LGB), and sexually diverse adolescents (ie, youth who do not identify as LGB but whose sexual orientation does not align with heterosexual norms [1]) report increased rates of sadness, hopelessness, suicide thoughts and behaviors, and poor mental health compared to heterosexual peers [2,3]. Additionally, sexual minority youth experience more severe symptoms of common yet treatable mental health disorders (eg, depression and generalized anxiety) than the general youth population [4]. Their elevated risk is associated with familial and social exclusion and chronic exposure to structural and interpersonal discrimination (eg, stigma, violence, and marginalization) [4-6], which is compounded for sexual minority youth of color who experience intersectional marginalization based on both sexual orientation and race and ethnicity [7]. Despite a greater need, sexual minority youth face significant barriers to care. In the United States, there is limited availability of affirmative providers for sexual minority youth. Research shows that only 28% of youth-serving mental health facilities offer services specifically for sexual minority youth. Further, availability is concentrated in coastal states and has not grown over time alongside increasing sexual minority youth population rates [8]. Barriers related to anticipated and enacted health care discrimination, cost, confidentiality, and parental consent concerns also restrict sexual minority youth’s access to needed mental health services [9-11]. These barriers are exacerbated for sexual minority youth of color who may face greater provider-based and treatment-seeking stigma, or receive inadequate or inappropriate care, including reduced access to culturally and linguistically appropriate services [12,13].

The COVID-19 pandemic saw both the onset of increasing rates of new mental health concerns [14] and deterioration of existing mental health conditions [15]. Research published since the start of COVID-19 indicates that compared to heterosexual peers, sexual minorities felt greater negative mental health consequences from the pandemic [16] likely due to multilevel vulnerabilities, including individual-, interpersonal-, community-, and structural-level risks [17]. In particular, while COVID-19 containment strategies mitigated risk for illness, they simultaneously exacerbated structural vulnerabilities connected to adverse mental health among sexual minority youth, and especially sexual minority youth of color, by disrupting access to safe housing options, community supports, and mental health services [12,17,18]. For example, access barriers that existed before COVID-19 were compounded by emergent pandemic-specific barriers that restricted access to care in new ways (eg, provider cancellations or closures and fear of COVID-19 exposure) and further widened mental health disparities among sexual minority youth [12,19].

Leading up to the COVID-19 pandemic, digital health and digital mental health services were increasingly recognized as promising solutions to overcome barriers restricting marginalized populations’ access to care [20]. For sexual minority youth and racially or ethnically minoritized youth, acceptance of and engagement with digital mental health services may depend on careful attention to community involvement and tailored and culturally relevant content [21-23]. COVID-19, however, served as a critical turning point that necessitated a rapid shift toward digital service delivery [24]. Because many digital mental health tools were developed and scaled quickly, few prioritized digital inclusion and equitable access for diverse needs, preferences, and access barriers [25-28]. Consequently, this shift may have inadvertently widened intragroup disparities (eg, by intersections of sexual orientation, race and ethnicity, age, geography, socioeconomic status, mental health need, and internet or device availability) in accessing and benefiting from digital mental health [29-31].

### This Study

The rapid transition to digital mental health during the COVID-19 pandemic has made it challenging to understand the digital divide, including varied access across different populations. This cross-sectional study identified the prevalence of digital mental health service use among US adolescents during the COVID-19 pandemic. Estimates of digital health service use were also explored as pediatricians increasingly provided mental health care during the COVID-19 pandemic [32]. An examination of heterogeneity by sexual orientation, race and ethnicity, and their intersection identified digital service use disparities among subpopulations of adolescents to inform targeted strategies to expand equitable access to digital health and digital mental health supports. This study was conducted following the STROBE (Strengthening the Reporting of Observational Studies in Epidemiology) reporting guidelines for cross-sectional studies (see Multimedia Appendix 1) [33].

## Methods

### Data and Participants

This cross-sectional study used data from the Adolescent Behaviors and Experiences Survey (ABES), a 110-item online questionnaire administered by the Centers for Disease Control and Prevention (CDC) from January to June 2021 [34]. The ABES was a one-time, probability-based survey of US high school students in grades 9-12 that assessed students’ health behaviors and experiences during the COVID-19 pandemic. The ABES adapted the methodology from the national Youth Risk Behavior Survey (YRBS), using the same stratified, 3-stage cluster sampling design to identify a nationally representative sample. A larger sample was drawn for the ABES, however, in anticipation of lower response rates during the COVID-19 pandemic. Weights based on student sex and grade accounted for school and student nonresponse and oversampling of Black students and Hispanic students. The school response rate was 37.8% (128/339), the student response rate was 48% (7705/16,037), and the overall response rate was 18% (38% * 48%). Additional documentation and data for the ABES are publicly available [34-36].

Of the 7998 submitted questionnaires, 7705 were retained after CDC data editing with 293 records excluded because fewer than 20 questions had been answered [34]. In this study, analytic samples varied due to missing data (see Multimedia Appendix 2). Missing data were not statistically imputed. In all models, missing data were handled using listwise deletion, a common approach in CDC-authored ABES (eg, [3,37,38]) and YRBS reports (eg, [39,40]) and secondary analyses of CDC data. Although listwise deletion can introduce bias, prior methodological work showed that the ABES’s complex sampling design and poststratification weighting mitigated nonresponse bias [34]. Further, minimal impact on estimates is expected when missingness is below 10% in secondary analyses of large surveys [41]. In this study, missingness ranged from 0.2% to 8.8%. Information on the number of participants who did not provide responses to study variables is provided in the relevant tables. Estimates were not reported when the unweighted denominator was <30, per CDC suppression guidance for the ABES [34,36].

### Analysis Plan

Table 1 presents the study measures. All analyses were conducted in Stata version 18.5 and were survey-adjusted using the “svy” command to account for the ABES' complex sampling design and weighting procedures. Distributions of participant characteristics were calculated for the full sample using unweighted frequencies and weighted percentages. Distribution and distributional differences in participant characteristics were also calculated by sexual orientation, by race and ethnicity, and by their intersection, with differences compared using Rao-Scott corrected chi-square tests for complex survey data. Weighted prevalence of digital service use outcomes (ie, digital mental health use and digital health use) was estimated for (1) the overall sample, (2) all sexual minority youth participants, (3) each sexual orientation group (heterosexual, LGB, and sexually diverse), (4) each racial or ethnic group (White, Black or African American, Hispanic or Latino, Asian or Pacific Islander, multiracial, and American Indian or Alaska Native), and (5) intersectional subgroups defined by both sexual orientation and race and ethnicity. A series of modified Poisson regression models were conducted to directly estimate adjusted prevalence ratios (aPRs) of digital mental health and digital health use, an appropriate approach for binary outcomes in cross-sectional data [42-44]. For all models, the “svy” command computes robust standard errors with a Huber-White sandwich estimator. Models tested statistical differences (*P*≤.05; 95% CI calculated) in prevalence of digital service use outcomes across the overall sample, including comparisons between (1) all sexual minority youth and heterosexual participants, (2) sexual minority youth subgroups (ie, LGB and sexually diverse) and heterosexual participants, and (3) racially or ethnically minoritized and White participants. Stratified models identified heterogeneity in digital service use outcomes among intersectional subgroups defined by both sexual orientation and race and ethnicity. Throughout all stratified analyses, the same set of intersectional subgroups is used; however, reference groups differ across models. Stratified models separately compared outcomes within racial or ethnic groups by sexual orientation and within sexual orientation groups by race and ethnicity by testing differences between (1) all sexual minority youth and heterosexual participants within racial and ethnic groups, (2) sexual minority youth subgroups and heterosexual participants within racial and ethnic groups, and (3) racially and ethnically minoritized and White participants within sexual orientation groups. These stratified models used Stata’s “subpop” command, which obtains accurate and unbiased estimates for analytic subpopulations while maintaining the full sample design information [45,46]. All regression analyses were adjusted for sex, age, mental health need, and internet access. Models comparing sexual orientation groups in the overall sample also adjusted for race and ethnicity.

**Table 1:** Description of study variables, questions, and analytic coding. Cross-sectional analysis of the Adolescent Behaviors and Experiences Survey (ABES), United States, January-June 2021.

Variables			Description
Sexual orientation			Participants selected one of the following: heterosexual (straight); gay or lesbian; bisexual; I describe my sexual identity some other way; I am not sure about my sexual identity (questioning); I do not know what this question is asking. ABES^a^ documentation further categorizes these selections into heterosexual; gay, lesbian, or bisexual; other or questioning; missing (ie, the aggregate of those who did not know what the question was asking (n=169) and those who skipped the question (n=372)) [34,36]. This study refers to the ABES category “other or questioning” as “sexually diverse,” reflecting inclusive terminology suggested by the National Academies of Engineering, Sciences, and Medicine [1].
Race and ethnicity			Participants indicated their race and ethnicity (ie, Hispanic heritage) by responding to 2 questions. Aligning with prior CDC^b^ data analyses [34,47-49], participants were categorized as non-Hispanic White (White), non-Hispanic Black or African American (Black or African American), Hispanic or Latino, non-Hispanic Asian or Pacific Islander (Asian unweighted, n=350; Native Hawaiian or other Pacific Islander unweighted, n=31), non-Hispanic multiracial (multiracial), and American Indian or Alaska Native.
**Digital service use indicators**			
	Digital mental health use	Participants indicated (no/yes) if they received mental health care, including treatment or counseling for substance use, using a computer, phone, or other device during the COVID-19 pandemic.	
	Digital health use	Participants indicated (no/yes) if they received medical care using a computer, phone, or other device during the COVID-19 pandemic.	
**Access indicators**			
	Mental health needs	Participants reported how often their mental health was not good during the COVID-19 pandemic. On the ABES questionnaire, poor mental health was defined parenthetically as including stress, anxiety, and depression. Responses included never, rarely, sometimes, most of the time, and always. ABES documentation dichotomizes responses as most of the time or always (1) and never, rarely, or sometimes (0) [36].	
	Device or internet access	Data were collected from schools at the time of recruitment for participation in ABES. Schools indicated (no/yes) if they provided laptops or Google Chromebooks, tablets, or Wi-Fi hotspots for students. Dichotomized as any access (1) and no access (0) to school-provided laptop, tablet, or Wi-Fi.	
	Background characteristics	*Sex* was reported as male or female. *Age* was reported in years, including those ages 14 years and under, 15, 16, 17, and 18 and older.	

^a^ABES: Adolescent Behaviors and Experiences Survey.

^b^CDC: US Centers for Disease Control and Prevention.

### Ethical Considerations

This secondary data analysis used publicly available, deidentified data and did not require approval by the University of Washington’s institutional review board in accordance with the US Common Rule (45 CFR 46 Subpart A). The original ABES study protocol was reviewed and approved by institutional review boards at the CDC and ICF International, the CDC’s survey contractor (45 CFR part 46; 21 CFR part 56). Parental permission and student assent were obtained during primary data collection. No additional consent was required for secondary analysis [34].

## Results

### Sample Characteristics

Table 2 displays the distribution of sample characteristics for the sample. Overall, participants were majority heterosexual, about half White, and evenly distributed across sex. About 37% (n=2643) of the sample indicated a mental health need during the COVID-19 pandemic, while the overwhelming majority reported access to a school-provided device or internet hotspot. (All percentages reported are weighted estimates.) Multimedia Appendix 3 displays the distribution of sample characteristics and uses heatmapping to visualize and identify distributive patterns by sexual orientation, by race and ethnicity, and by their intersection. LGB and sexually diverse participants, for example, were predominantly female and reported greater mental health need than heterosexual participants (*P*≤.05). Elevated mental health need was also identified among White, Hispanic or Latino, and multiracial participants (*P*≤.05). Within most racial or ethnic groups, significantly higher rates of poor mental health were observed among sexual minority youth compared to heterosexual participants of the same race and ethnicity. Strikingly, LGB and sexually diverse multiracial participants reported poor mental health at rates close to 69% (n=55) and 76% (n=34), respectively.

**Table 2:** Unweighted frequencies and weighted prevalence of participant sociodemographic characteristics (N=7750)^a^. Cross-sectional analysis of the Adolescent Behaviors and Experiences Survey (ABES), United States, January-June 2021.

			Values, n (%)
**Sexual orientation**			
	Heterosexual	5539 (77.5)	
	LGB^b^	977 (13.2)	
	Sexually diverse	648 (9.3)	
**Race and ethnicity**			
	White	3461 (49.8)	
	Black or African American	1189 (12.9)	
	Hispanic or Latino	2038 (25.4)	
	Asian or Pacific Islander	381 (5.5)	
	Multiracial (non-Hispanic)	480 (5.8)	
	American Indian or Alaska Native	83 (0.7)	
**Sex**			
	Male	3678 (49.6)	
	Female	3999 (50.4)	
**Age (years)**			
	≤14	914 (11.8)	
	15	1933 (24.4)	
	16	1971 (25.3)	
	17	1804 (24.2)	
	≥18	1070 (14.3)	
Mental health needs, yes			2643 (37.1)
Device or internet access, yes			7548 (97.7)

^a^All percentages are valid percentages, calculated based on the number of participants who reported data for each category. The number of respondents who did not provide information about demographic characteristics or access indicators was as follows: sexual orientation (n=541), race and ethnicity (n=73), sex (n=28), age (n=13), mental health need (n=498), and device or internet access (n=33).

^b^LGB: lesbian, gay, and bisexual.

### Digital Mental Health Use

**Figure 1:**
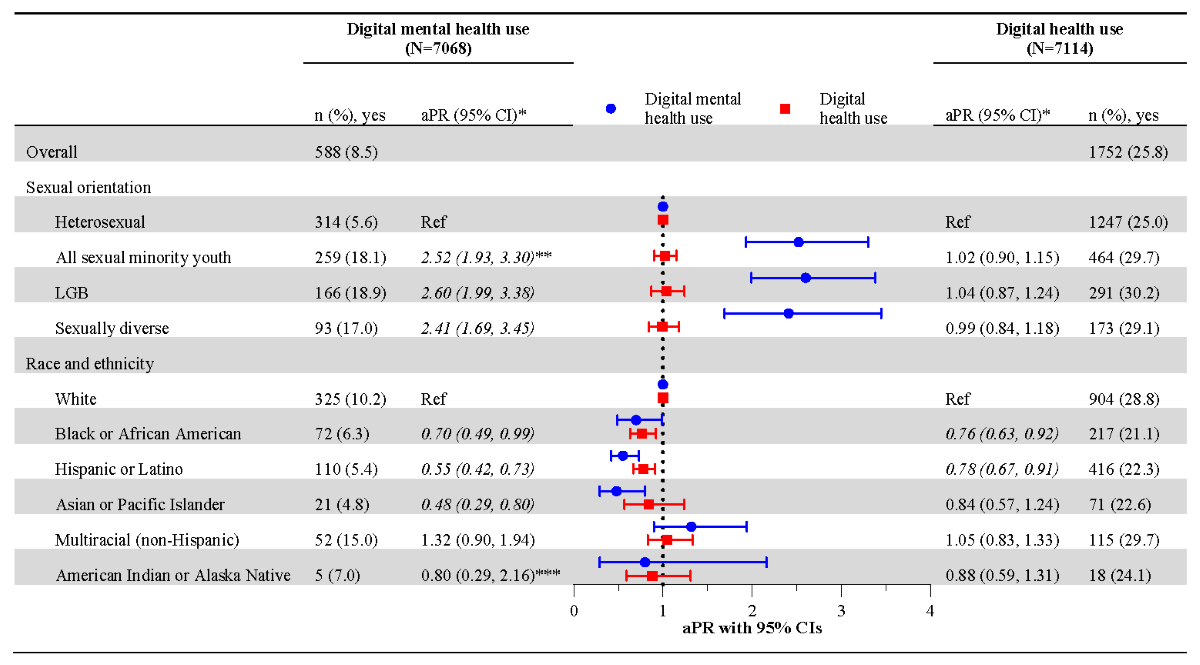
Unweighted frequencies, weighted unadjusted prevalence, and adjusted prevalence ratios of digital mental health and digital health service use by sexual orientation and by race and ethnicity. Cross-sectional analysis of the Adolescent Behaviors and Experiences Survey (ABES), United States, January-June 2021. aPR: adjusted prevalence ratio; LGB: lesbian, gay, and bisexual. *Estimates adjusted for sex, age, mental health need, and device or internet access. Sexual orientation estimates were also adjusted for race and ethnicity. **Italicized outcomes indicate differences at *P*<.05. ***Estimate is based on the occurrence of ≤10 unweighted events of digital mental health use and should be interpreted with caution.

Prevalence of digital mental health use was 8.5% (n=588) across the sample, with estimates varying by sexual orientation and race and ethnicity (Figure 1). Digital mental health use was 5.6% (n=314) among heterosexual participants and 18.1% (n=259) among all sexual minority youth. (All percentages reported are weighted estimates.) In adjusted models examining the association of sexual orientation and digital mental health use, all sexual minority youth had a higher prevalence of digital mental health use compared to heterosexual participants (aPR 2.52, 95% CI 1.93-3.30). This pattern was consistent across both sexual minority youth subgroups: LGB (aPR 2.60, 95% CI 1.99-3.38) and sexually diverse participants (aPR 2.41, 95% CI 1.69-3.45). Digital mental health use was 10.2% among White participants (n=325) and ranged from 4.8% to 15% among racially and ethnically minoritized participants (see Figure 1). In adjusted models examining the association of race and ethnicity and digital mental health use, Black or African American (aPR 0.70, 95% CI 0.49-0.99), Hispanic or Latino (aPR 0.55, 95% CI 0.42-0.73), and Asian or Pacific Islander participants (aPR 0.48, 95% CI 0.29-0.80) had lower prevalence of digital mental health use relative to White peers.

**Figure 2:**
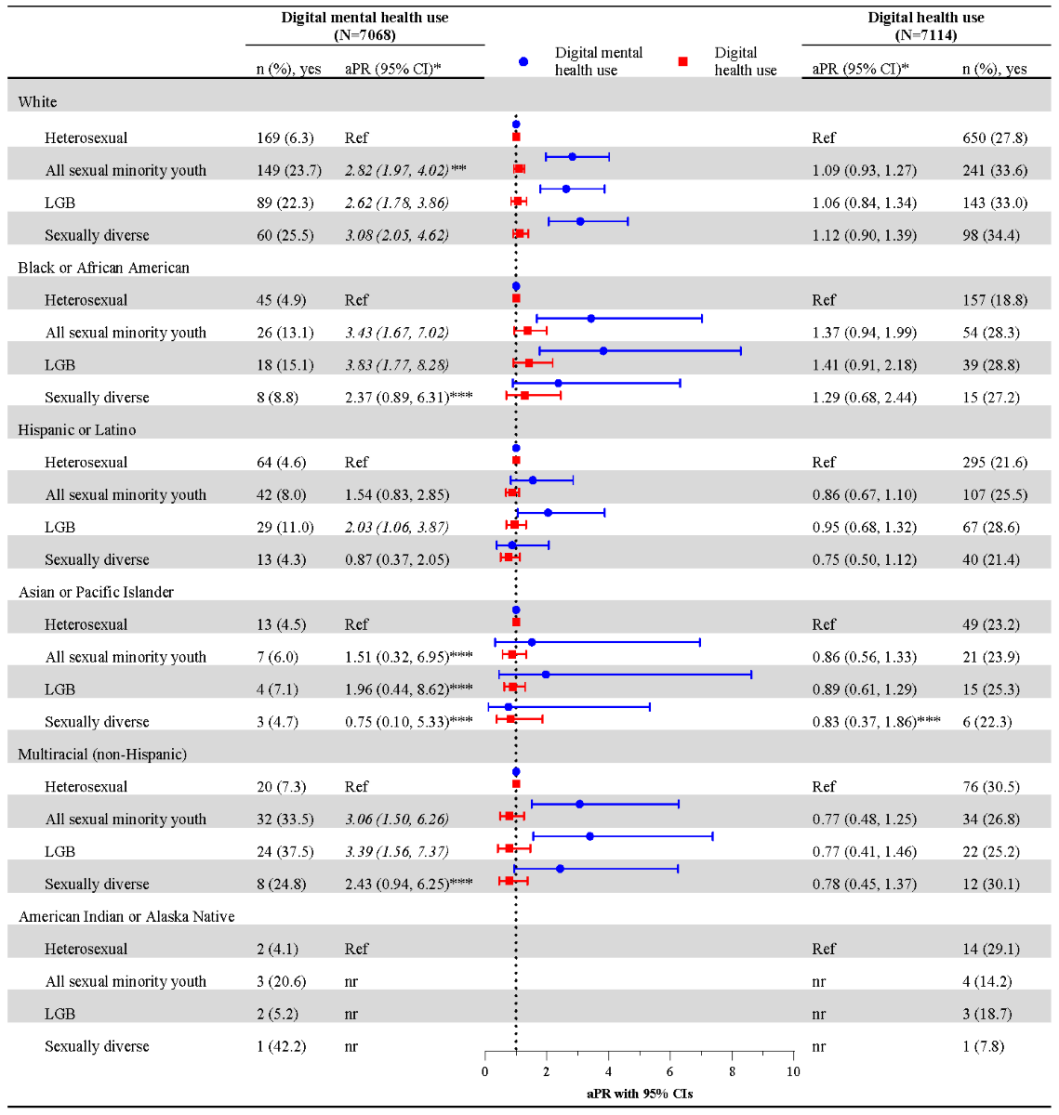
Unweighted frequencies, weighted unadjusted prevalence, and adjusted prevalence ratios of digital mental health and digital health service use within racial and ethnic subgroups by sexual orientation. Cross-sectional analysis of the Adolescent Behaviors and Experiences Survey (ABES), United States, January-June 2021. aPR: adjusted prevalence ratio; LGB: lesbian, gay, and bisexual; nr: not reported because the unweighted denominator (n) is <30, per CDC suppression guidance for ABES. *Estimates adjusted for sex, age, mental health need, and device or internet access. **Italicized outcomes indicate differences at *P*<.05. ***Estimate is based on the occurrence of ≤10 unweighted events of digital mental health use or digital health use and should be interpreted with caution.

Figure 2 examines digital mental health use among intersectional subgroups, comparing the prevalence of digital mental health use across sexual orientation within each racial or ethnic group, using heterosexual participants as the reference. White, Black or African American, and multiracial sexual minority youth all reported a higher prevalence of digital mental health use compared to heterosexual peers. Among White participants, 6.3% (n=169) of heterosexual youth used digital mental health compared to 23.7% of all sexual minority youth (aPR 2.82). Similar differences were observed among Black or African American (all sexual minority youth: 13.1% vs heterosexual: 4.9%; aPR 3.43) and multiracial participants (all sexual minority youth: 33.5% vs heterosexual: 7.3%; aPR 3.06). These patterns were consistent across White, Black or African American, and multiracial LGB and sexually diverse subgroups relative to heterosexual peers (see Figure 2 for estimates). Among Hispanic or Latino participants, those who were LGB, specifically, had a higher prevalence of digital mental health use (11%) than heterosexual peers (4.6%; aPR 2.03). Asian or Pacific Islander sexual minority youth showed no differences compared to heterosexual peers despite heightened mental health needs.

**Figure 3:**
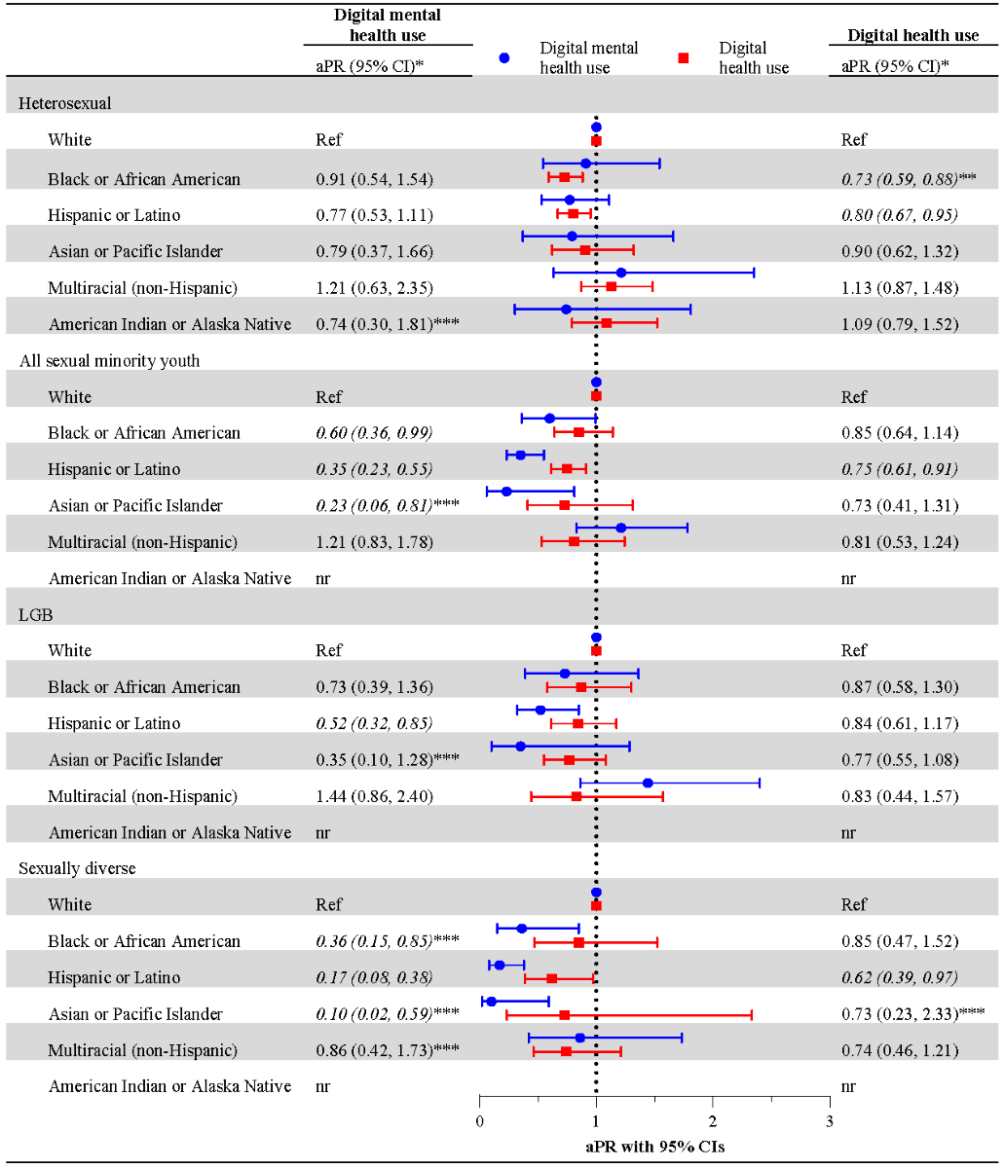
Adjusted prevalence ratios of digital mental health and digital health service use within sexual orientation subgroups by race and ethnicity. Cross-sectional analysis of the Adolescent Behaviors and Experiences Survey (ABES), United States, January-June 2021. aPR: adjusted prevalence ratio; nr: not reported because the unweighted denominator (n) is <30, per CDC suppression guidance for ABES. *Estimates adjusted for sex, age, mental health need, and device or internet access. **Italicized outcomes indicate differences at *P*<.05. ***Estimate is based on the occurrence of ≤10 unweighted events of digital mental health use or digital health use and should be interpreted with caution.

Figure 3 examines digital mental health use among intersectional subgroups, comparing prevalence of digital mental health use across race and ethnicity within each sexual orientation group, using White participants as the reference. These models highlighted racial or ethnic disparities within sexual orientation groups (see Figure 2 for prevalence of digital mental health use among intersectional subgroups and Figure 3 for aPRs and 95% CIs). Black or African American (13.1%; aPR 0.60), Hispanic or Latino (8%; aPR 0.35), and Asian or Pacific Islander sexual minority youth (6%; aPR 0.23) had lower prevalence of digital mental health than White sexual minority youth (23.7%). These same disparities persisted for sexually diverse subgroups, specifically. Black or African American (8.8%; aPR 0.36), Hispanic or Latino (4.3%; aPR 0.17), and Asian or Pacific Islander (4.7%; aPR 0.10) sexually diverse participants reported a lower prevalence of digital mental health use than White sexually diverse participants (25.5%). Racial or ethnic disparities were also pronounced for Hispanic or Latino (11%; aPR 0.52) LGB participants compared to White peers (22.3%). No differences in digital mental health use by race and ethnicity were identified among heterosexual participants.

### Digital Health Use

Across the sample, digital health use was 25.8% (n=1752) (Figure 1). (All percentages reported are weighted estimates.) No significant differences in digital health use were identified by sexual orientation, including in the overall sample or when stratified by race and ethnicity (see Figures 1 and 2, respectively). However, some racial or ethnic disparities in digital health use were observed when comparing across racial or ethnic groups, both in the overall sample and within sexual orientation groups. Across the sample, Black or African American (21%, aPR 0.76) and Hispanic or Latino (22.3%, aPR 0.78) participants reported a lower prevalence of digital health use than their White peers (28.8%) (Figure 1). Figure 3 shows that these disparities persisted within sexual orientation groups. Black or African American and Hispanic or Latino participants often reported lower digital health use compared to their White peers, including among heterosexual (Black or African American: 18.8%, aPR 0.73; Hispanic or Latino: 21.6%, aPR 0.80), all sexual minority youth (Hispanic or Latino: 25.5%, aPR 0.75), and sexually diverse participants (Hispanic or Latino: 21.4%, aPR 0.62). These participants had a lower prevalence of digital health use than their White heterosexual (27.8%), White sexual minority youth (33.6%), and White sexually diverse peers (34.4%), respectively.

### Sensitivity Analysis

While analyses controlled for device or internet access as a direct correlate of digital service use, this school-provided resource does not adequately capture key barriers or facilitators of digital mental health and digital health use. The ABES did not include direct measures of individual- or household-level correlates of digital service use. However, sensitivity analyses were conducted by including additional covariates in the tested models. Parental job loss or unemployment, English language proficiency, and housing instability (see Multimedia Appendix 4 for full descriptions of measures, including ABES survey questions) served as proxies for access barriers, for example, related to insurance or cost, language, and confidentiality or privacy (eg, through limited access to private spaces). Minimal changes to estimates were identified with the inclusion of these additional covariates. Generally, aPRs were slightly attenuated (see Multimedia Appendices 5-7 compared to Figures 1-3). Additionally, prevalence of digital mental health use was no longer significantly different among Black or African American and White participants overall (see Multimedia Appendix 5) or Black or African American and White sexual minority youth (see Multimedia Appendix 7).

## Discussion

### Principal Findings

This cross-sectional study examined digital mental health and digital health use among US youth during the COVID-19 pandemic, highlighting digital service use disparities at the intersection of sexual orientation and race and ethnicity. Sexual minority youth, overall, reported a higher prevalence of digital mental health use than heterosexual peers, potentially reflecting these youths’ connection to digital mental health prior to COVID-19 due to persistent barriers to in-person care, such as stigma, lack of affirming providers, and confidentiality concerns [9,11,12,50].

This pattern, however, obscures critical subgroup disparities among sexual minority youth of color whose navigation of and engagement with care, including digital services, is shaped by interlocking systems of marginalization (eg, racism and heterosexism) that create unique and compounding barriers. While digital service delivery offers improved accessibility for some sexual minority youth, especially those already engaged with digital mental health resources before the pandemic [12], the persistent racial or ethnic disparities identified in this study suggest that the availability of digital services alone is insufficient. Like in-person care, these services must be both offered and perceived as a viable option [10]. Yet, structural and cultural barriers (eg, trust, relevance, and representation) remain and may limit engagement even when accounting for access to infrastructure factors (eg, internet or device availability) [51-53]. In this study, Black or African American, Hispanic or Latino, and Asian or Pacific Islander sexual minority youth, particularly sexually diverse youth, had a lower prevalence of digital mental health use than their White sexual minority youth counterparts. These results align with broader concerns that rapid digital mental health scaling during the COVID-19 pandemic exacerbated digital inequities by overlooking the needs of multiply marginalized populations [27]. Many digital mental health resources remain inadequately tailored to sexual minority youth of color [54], and without intentional inclusion (eg, cocreation with lived-experience experts through each step of design, development, and evaluation [55,56]), these services risk reinforcing existing inequities. Incorporating end-user feedback early and consistently can promote innovation and may improve acceptability and usability through enhanced cultural and contextual relevance. For sexual minority youth of color, specifically, the cocreation process must be inclusive and attuned to power differentials for successful digital mental health development (eg, foster safety for sexual minority youth of color to discuss their experiences, needs, and preferences) [55]. Without cocreation, clinically promising tools may ultimately leave behind sexual minority youth of color by failing to adequately address their needs. However, when meaningfully implemented, cocreation methods can garner trust and help work toward building digital mental health services that reflect the lived realities and meet the needs of diverse users [28,55,56].

Digital health use did not differ between heterosexual and sexual minority youth, suggesting identity-based factors may be less salient in this context. These findings may imply that digital health services may be more normalized, broadly accessible, or perceived as less stigmatizing than digital mental health services. Accordingly, they may offer an important point of access for addressing mental health concerns, particularly for youth who may not seek or trust digital mental health services. Still, persistent racial or ethnic digital health disparities within both heterosexual and sexual minority youth subgroups highlight possible structural inequities that drive these disparities. For example, despite similar rates of mental health need as White participants, Hispanic or Latino participants overall and Hispanic or Latino sexual minority youth, specifically, had a lower prevalence of digital health use. During the height of COVID-19, disrupted family dynamics and resources (eg, due to bereavement, job loss, and distance learning), limited privacy due to crowded living environments and shared device use, and amplified access challenges related to mistrust or misinformation, digital literacy, and language barriers disproportionately impacted racially or ethnically minoritized groups, especially Black and Latino populations [57,58]. To close access gaps, digital health services must engage in targeted outreach that communicates both the availability and value of culturally responsive and affirming care created in collaboration with diverse youth [52,57].

### Limitations

Limitations must be considered when interpreting findings. Due to the cross-sectional nature of the ABES data, results do not imply causality. This study is also limited by small subgroup sizes and grouping a range of identities within LGB and sexually diverse categories due to sample size. Some intersectional subgroup estimates were suppressed or had wide confidence intervals (eg, Asian or Pacific Islander and American Indian or Alaska Native sexual minority youth subgroups), limiting the interpretability of these comparisons. Further, despite guidance for combining Asian or Pacific Islander participants into a single racial or ethnic category, this approach may erase important differences that are associated with health and health care outcomes (eg, cultural approaches to care, histories of colonization, and language access) [59]. Additionally, while our analyses examined digital mental health and digital health use among intersectional subgroups defined by both sexual orientation and race and ethnicity, quantitative approaches inherently simplify the lived realities of multiply marginalized populations, including sexual minority youth of color. Heterosexism and racism, among other forms of marginalization (eg, cissexism, ableism, and classism), are multiplicatively experienced, interacting in nuanced ways to shape how youth perceive, seek, and experience care [60]. Future research may consider qualitative or mixed methods to capture these complexities to guide the development of digital services.

Because of the broad behavioral domains assessed by the ABES, many correlates specific to digital service use were not captured in the current analyses. While some access factors (ie, mental health need and device or internet access) were examined, only proxy measures of additional access factors could be included in sensitivity analyses. This exclusion may result in residual-confound disparities due to unmeasured or inadequately measured individual-, household-, or system-level access factors. Finally, the service use measures relied on self-reported data rather than administrative health records. Preliminary evaluation of missingness suggested data met missing at random assumptions conditional on observed demographic variables. However, it is possible that unmeasured factors such as mental health stigma and social desirability may bias service use estimates given the sensitive nature of mental health and other ABES topics [61]. Further, these measures captured whether participants accessed services but not the care type (eg, self-guided, provider-guided, app-based, and telehealth), quality, frequency, or adequacy. This measurement limitation may obscure meaningful differences in how youth actually engaged with services. For example, a binary indicator cannot distinguish between a single, brief interaction versus ongoing, structured care with human support, the latter of which evidence indicates is effective with robust rates of treatment response [62]. As such, this study may understate or overstate the impact of disparities by failing to capture variation in digital mental health and digital health relevance, satisfaction, or effectiveness. More nuanced and multidimensional measures are needed to assess disparities in meaningful and sustained access to care [63].

### Conclusions

Despite limitations, this study offers critical insight into how digital mental health and digital health use vary across intersecting identities. Results suggest digital services may help reduce some barriers to care, especially for sexual minority youth already familiar with online resources prior to the pandemic. Persistent racial or ethnic disparities, however, reflect broader systemic inequities that digital solutions alone cannot resolve. Addressing digital exclusion and bridging the digital divide will require more than expanding availability; it will demand digital mental health resources that are cocreated through participatory and iterative processes where the voices of multiply marginalized youth directly inform the design and delivery of care. Digital mental health must be inclusive, culturally grounded, and clinically relevant to truly advance equitable care for all youth.

## Appendix

Multimedia Appendix 1 STROBE reporting guidelines for cross-sectional studies.

Multimedia Appendix 2 Participant flow diagram for sexual orientation, race and ethnicity, and intersectional subgroup models. Cross-sectional analysis of the Adolescent Behaviors and Experiences Survey (ABES), United States, January-June 2021.

Multimedia Appendix 3 Unweighted frequencies and weighted prevalence of participant sociodemographic characteristics by sexual orientation, by race and ethnicity, and by intersectional subgroup (N=7750). Cross-sectional analysis of the Adolescent Behaviors and Experiences Survey (ABES), United States, January-June 2021.

Multimedia Appendix 4 Description of access proxy variables, questions, analytic coding, unweighted frequencies, and weighted prevalence for sensitivity analysis. Cross-sectional analysis of the Adolescent Behaviors and Experiences Survey (ABES), United States, January-June 2021.

Multimedia Appendix 5 Sensitivity analysis, adjusted prevalence ratios (aPRs) of digital mental health and digital health service use by sexual orientation and by race and ethnicity. Cross-sectional analysis of the Adolescent Behaviors and Experiences Survey (ABES), United States, January-June 2021.

Multimedia Appendix 6 Sensitivity analysis, adjusted prevalence ratios (aPRs) of digital mental health and digital health service use within racial and ethnic subgroups by sexual orientation. Cross-sectional analysis of the Adolescent Behaviors and Experiences Survey (ABES), United States, January-June 2021.

Multimedia Appendix 7 Sensitivity analysis, adjusted prevalence ratios (aPRs) of digital mental health and digital health service use within sexual orientation subgroups by race and ethnicity. Cross-sectional analysis of the Adolescent Behaviors and Experiences Survey (ABES), United States, January-June 2021.

Multimedia Appendix 8 Stata syntax.

## Abbreviations

ABES: Adolescent Behaviors and Experiences Survey
aPR: adjusted prevalence ratio
CDC: Centers for Disease Control and Prevention
LGB: lesbian, gay, and bisexual
STROBE: Strengthening the Reporting of Observational Studies in Epidemiology
YRBS: Youth Risk Behavior Survey

## Data Availability

Documentation and data for the ABES are publicly available [35]. Stata syntax for the study analysis can be found in Multimedia Appendix 8.
