# Supplementary material for: Intersectional Disparities in Digital Health and Mental Health Service Use Among US Youth During the COVID-19 Pandemic: Cross-Sectional Analysis of a National Survey
Source: J Med Internet Res. 2025 Oct 27;27:e77062. doi: 10.2196/77062 (PMC12603589; doi:10.2196/77062)
Supplement: Multimedia Appendix 3 [file jmir_v27i1e77062_app3.docx]

| **Multimedia Appendix 3.** Unweighted frequencies and weighted prevalence of participant sociodemographic characteristics by sexual orientation, by race and ethnicity, and by intersectional subgroup (N=7750).^a^ Cross-sectional analysis of the Adolescent Behaviors and Experiences Survey (ABES), United States, January-June 2021. | | | | | | | | | | | | | | | | | | | | |
| --- | --- | --- | --- | --- | --- | --- | --- | --- | --- | --- | --- | --- | --- | --- | --- | --- | --- | --- | --- | --- |
|  |  | | **Total sample** | **Sex** | | | | **Age** | | | | | | | **Mental health need** | | | **Device or internet access** | | |
|  |  | | n^b^ | Male | Female | F  (df1, df2)^c^ | *P* value | ≤ 14 years | 15 years | 16 years | 17 years | ≥ 18 years | F  (df1, df2)^c^ | *P* value | yes | F  (df1, df2)^c^ | *P*  value | yes | F  (df1, df2)^c^ | *P* value |
| Sexual orientation,  n (%) | | |  |  |  | 99.1  (2,108) | ≤0.001 |  |  |  |  |  | 1.9  (6.4, 346.9) | 0.076 |  | 90.2  (1.7, 93.4) | ≤0.001 |  | 0.7  (1, 55.8) | 0.257 |
|  | Heterosexual | | 5539 | 3055 (56.9) | 2481 (43.1) |  |  | 647 (11.7) | 1393 (24.1) | 1409 (25.1) | 1296 (23.9) | 789 (15.3) |  |  | 1594 (30.3) |  |  | 5445 (98.0) |  |  |
|  | LGB | | 977 | 200 (21.6) | 770 (78.4) |  |  | 113 (10.9) | 258 (24.7) | 260 (26.1) | 220 (25.8) | 125 (12.6) |  |  | 612 (63.8) |  |  | 952 (97.2) |  |  |
|  | Sexually diverse | | 648 | 127 (21.9) | 508 (78.1) |  |  | 97 (15.6) | 174 (28.9) | 161 (23.3) | 147 (22.6) | 68  (9.6) |  |  | 395 (61.5) |  |  | 638 (98.3) |  |  |
| Race and ethnicity,  n (%) | | |  |  |  | 0.5  (3.3, 176.3) | 0.684 |  |  |  |  |  | 2.0  (8.1, 436.2) | 0.045 |  | 7.2  (4, 217) | ≤0.001 |  | 0.8  (2, 106.2) | 0.429 |
|  | White | | 3461 | 1672 (50.5) | 1781 (49.5) |  |  | 399 (10.9) | 902 (24.6) | 877 (24.8) | 794 (23.6) | 486 (16.1) |  |  | 1309 (40.2) |  |  | 3384 (97.4) |  |  |
|  | Black or African American | | 1189 | 589 (50.7) | 595 (49.3) |  |  | 137 (13.5) | 291 (23.1) | 314 (25.8) | 274 (23.9) | 173 (13.7) |  |  | 289 (28.0) |  |  | 1151 (95.4) |  |  |
|  | Hispanic or Latino | | 2038 | 921 (47.3) | 1111 (52.7) |  |  | 263 (14.4) | 500 (25.7) | 487 (24.6) | 510 (24.7) | 275 (10.6) |  |  | 714 (36.8) |  |  | 2021 (99.2) |  |  |
|  | Asian or Pacific Islander | | 381 | 175 (49.6) | 204 (50.5) |  |  | 47 (11.2) | 93  (19.8) | 102 (26.9) | 93  (28.6) | 44 (13.5) |  |  | 124 (32.0) |  |  | 363 (96.7) |  |  |
|  | Multiracial | | 480 | 238 (48.2) | 239 (51.8) |  |  | 53 (6.7) | 110 (21.8) | 144 (27.9) | 110 (27.2) | 63  (16.4) |  |  | 171 (40.0) |  |  | 473 (98.3) |  |  |
|  | American Indian or Alaska Native | | 83 | 40 (50.8) | 43 (49.2) |  |  | 8 (7.3) | 17  (24.9) | 25  (33.7) | 12  (13.4) | 21  (20.7) |  |  | 17  (23.3) |  |  | 83  (100) |  |  |
| Intersectional Subgroups, n (%) | | |  |  |  |  |  |  |  |  |  |  |  |  |  |  |  |  |  |  |
|  | White | |  |  |  | 40.0  (2, 106.1) | ≤0.001 |  |  |  |  |  | 2.0  (4.7, 252.7) | 0.077 |  | 41.2  (1.8, 98.2) | ≤0.001 |  | 1.1  (1, 56) | 0.295 |
|  |  | Heterosexual | 2549 | 1421 (57.6) | 1128 (42.4) |  |  | 292 (10.9) | 674 (24.6) | 610 (23.6) | 581 (23.0) | 390 (18.0) |  |  | 815 (33.7) |  |  | 2508 (98.2) |  |  |
|  |  | LGB | 441 | 98 (24.7) | 340 (75.3) |  |  | 50 (9.5) | 103 (21.1) | 140 (30.2) | 95  (26.0) | 53  (13.3) |  |  | 301 (65.7) |  |  | 432 (96.8) |  |  |
|  |  | Sexually diverse | 289 | 65 (26.9) | 220 (73.1) |  |  | 39 (14.1) | 81  (28.4) | 74  (25.0) | 70  (23.7) | 25  (8.8) |  |  | 180 (63.4) |  |  | 283 (97.9) |  |  |
|  | Black or African American | |  |  |  | 32.2  (1.7, 90.2) | 0.077 |  |  |  |  |  | 2.5  5.6, 301.4) | 0.028 |  | 21.9  1.9, 100.2) | ≤0.001 |  | 3.2  (1.1, 57.8) | 0.078 |
|  |  | Heterosexual | 851 | 478 (57.0) | 372 (43.0) |  |  | 93 (12.8) | 206 (22.3) | 230 (25.8) | 199 (24.7) | 123 (14.4) |  |  | 172 (21.8) |  |  | 822 (94.5) |  |  |
|  |  | LGB | 154 | 27 (15.7) | 127 (84.4) |  |  | 23 (20.0) | 44  (29.7) | 33  (23.1) | 31  (15.3) | 23  (11.9) |  |  | 77  (54.7) |  |  | 149 (96.5) |  |  |
|  |  | Sexually diverse | 69 | 13 (11.4) | 54 (88.6) |  |  | 10 (14.8) | 22  (41.8) | 23  (27.3) | 11  (14.0) | 3  (2.1) |  |  | 35  (53.2) |  |  | 68  (98.4) |  |  |
|  | Hispanic or Latino | |  |  |  | 55.2  (1.6, 88.5) | ≤0.001 |  |  |  |  |  | 1.2  (4.3, 232.7) | 0.323 |  | 46.2  (1.9, 100.7) | ≤0.001 |  | 0.7  (1.1, 58.8) | 0.419 |
|  |  | Heterosexual | 1456 | 773 (55.1) | 683 (44.9) |  |  | 186 (14.2) | 357 (25.3) | 363 (25.1) | 362 (24.6) | 188 (10.9) |  |  | 430 (29.4) |  |  | 1448 (99.4) |  |  |
|  |  | LGB | 237 | 41 (19.2) | 194 (80.8) |  |  | 22 (10.2) | 66  (29.4) | 48  (20.0) | 67  (30.8) | 34  (9.6) |  |  | 148 (66.6) |  |  | 230 (98.0) |  |  |
|  |  | Sexually diverse | 191 | 29 (15.7) | 159 (84.3) |  |  | 31 (19.1) | 48  (29.0) | 39  (24.4) | 47  (19.1) | 26  (8.4) |  |  | 118 (61.4) |  |  | 191 (100) |  |  |
|  | Asian or PI | |  |  |  | 18.4  (1.7, 91.1) | ≤0.001 |  |  |  |  |  | 1.0  (4.1, 221.9) | 0.437 |  | 12.3  (2, 105.8) | ≤0.001 |  | 1.1  (1, 55.7) | 0.293 |
|  |  | Heterosexual | 260 | 138 (58.9) | 122 (41.1) |  |  | 33 (11.1) | 61  (18.6) | 75  (26.3) | 62  (26.3) | 28  (14.0) |  |  | 72  (26.4) |  |  | 426 (97.0) |  |  |
|  |  | LGB | 45 | 8 (20.4) | 37 (79.6) |  |  | 8 (16.4) | 13  (20.8) | 10  (23.9) | 9  (30.1) | 4  (8.9) |  |  | 26  (54.4) |  |  | 44  (98.0) |  |  |
|  |  | Sexually diverse | 44 | 7 (11.1) | 35 (88.8) |  |  | 5 (11.2) | 15  (34.3) | 11  (11.6) | 10  (32.1) | 3  (10.8) |  |  | 23  (48.1) |  |  | 41  (92.0) |  |  |
|  | Multiracial | |  |  |  | 8.3  (1.9, 101.2) | 0.0006 |  |  |  |  |  | 1.4  (5.3, 283.8) | 0.229 |  | 22.2  (1.9, 104.2) | ≤0.001 |  | 0.5  (1, 54.8) | 0.472 |
|  |  | Heterosexual | 324 | 187 (56.0) | 137 (44.0) |  |  | 33 (5.8) | 73  (22.8) | 97  (28.7) | 78  (28.0) | 43  (14.8) |  |  | 81  (27.3) |  |  | 322 (99.2) |  |  |
|  |  | LGB | 85 | 21 (20.0) | 62 (80.0) |  |  | 10 (5.3) | 26  (24.5) | 24  (24.5) | 16  (24.8) | 9  (21.0) |  |  | 55  (69.1) |  |  | 82  (97.6) |  |  |
|  |  | Sexually diverse | 41 | 10 (32.5) | 30 (67.5) |  |  | 9 (19.6) | 6  (11.6) | 11  (14.7) | 7  (28.9) | 8  (25.2) |  |  | 34  (75.9) |  |  | 41  (100) |  |  |
|  | American Indian or Alaska Native | |  |  |  | 1.6  (1.4, 37.8) | 0.213 |  |  |  |  |  | 1.7  (2.1, 56.2) | 0.19 |  | 1.2  (1.6, 43.4) | 0.306 |  | ―^d^ | ―^d^ |
|  |  | Heterosexual | 56 | 30 (52.4) | 26 (47.6) |  |  | 7 (10.5) | 11  (22.7) | 19  (39.2) | 7  (10.6) | 12  (17.0) |  |  | 11  (19.6) |  |  | 56  (100) |  |  |
|  |  | LGB | 11 | 3 (27.8) | 8 (72.2) |  |  | 0 (0) | 4  (56.7) | 3  (15.9) | 2  (7.4) | 2  (28.5) |  |  | 4  (43.3) |  |  | 11  (100) |  |  |
|  |  | Sexually diverse | 6 | 1 (26.4) | 5 (73.6) |  |  | 0 (0) | 1  (31.1) | 1  (8.3) | 2  (28.5) | 2  (32.1) |  |  | 2  (53.5) |  |  | 6  (100) |  |  |
| 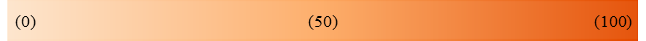Notes: | | | | | | | | | | | | | | | | | | | | |
| a. All percentages are valid percent, calculated based on the number of participants who reported data for each category. The number of respondents who did not provide information about demographic characteristics or access indicators were as follows: sexual orientation (n=541), race and ethnicity (n=73), sex (n=28), age (n=13), mental health need (n=498), device or internet access (n=33). | | | | | | | | | | | | | | | | | | | | |
| b. Unweighted frequency. | | | | | | | | | | | | | | | | | | | | |
| c. Rao-Scott corrected chi-square tests. ꭓ^2^ statistic corrected for complex survey design (ie, design-based F). | | | | | | | | | | | | | | | | | | | | |
| d. Not computed due to no variation in responses. | | | | | | | | | | | | | | | | | | | | |
