# Supplementary material for: Intersectional Disparities in Digital Health and Mental Health Service Use Among US Youth During the COVID-19 Pandemic: Cross-Sectional Analysis of a National Survey
Source: J Med Internet Res. 2025 Oct 27;27:e77062. doi: 10.2196/77062 (PMC12603589; doi:10.2196/77062)
Supplement: Multimedia Appendix 4 [file jmir_v27i1e77062_app4.docx]

| **Multimedia Appendix 4.** Description of access proxy variables, questions, analytic coding, unweighted frequencies, and weighted prevalence for sensitivity analysis. Cross-sectional analysis of the Adolescent Behaviors and Experiences Survey (ABES), United States, January-June 2021. | | |  |
| --- | --- | --- | --- |
| **Variables** | | **Description** | **n (%)^a^** |
| *Access Proxies* | |  |  |
|  | Parental job loss or unemployment | Participants were asked, "During the COVID-19 pandemic, did a parent or other adult in your home lose their job even for a short amount of time?" Parental job loss/unemployment was indicated for those who responded *yes* or *parents did not have jobs before the COVID-19 pandemic* (1) and not indicated for those who responded *no* (0). | 2216 (31.2) |
|  | English language proficiency | Participants were asked, "How well do you speak English?" Per ABES guidance, proficiency was indicated for those who responded *very well* or *well* (1) and not indicated for those who responded *not well* or *not at all* (0). | 6975 (98.0) |
|  | Housing instability | Participants were asked, "During the past 30 days, where did you usually sleep?" Per ABES guidance, housing instability was indicated for those who responded *in the home of a friend*, *family member, or other person because I had to leave my home*; *my parent or guardian cannot afford housing*; *in a shelter or emergency housing*; *in a motel or hotel*; *in a car, park, campground, or other public place*; *I do not have a usual place to sleep*, or; *somewhere else* (1). Housing instability was not indicated for those who responded *in my parent's or guardian's home* (0). | 244 (3.1) |
| Notes: | | | |
| a. Unweighted frequencies and weighted prevalence. All percentages are valid percent, calculated based on the number of participants who reported data for each category. The number of respondents who did not provide information about access proxies were as follows: parental job loss or unemployment (n=526), English language proficiency (n=580), housing instability (n=635). | | | |
