# Supplementary material for: Intersectional Disparities in Digital Health and Mental Health Service Use Among US Youth During the COVID-19 Pandemic: Cross-Sectional Analysis of a National Survey
Source: J Med Internet Res. 2025 Oct 27;27:e77062. doi: 10.2196/77062 (PMC12603589; doi:10.2196/77062)
Supplement: Multimedia Appendix 5 [file jmir_v27i1e77062_app5.docx]

| **Multimedia Appendix 5.** Sensitivity analysis, adjusted prevalence ratios (aPRs) of digital mental health and digital health service use by sexual orientation and by race and ethnicity. Cross-sectional analysis of the Adolescent Behaviors and Experiences Survey (ABES), United States, January-June 2021. | | | | |
| --- | --- | --- | --- | --- |
|  |  | **Digital mental health service use^a^** |  | **Digital health service use^a^** |
|  | | aPR (95% CI)^bc^ |  | aPR (95% CI)^bc^ |
| **Sexual orientation** | |  |  |  |
|  | Heterosexual | Ref |  | Ref |
|  | All sexual minority youth | **2.37 (1.81, 3.11)** |  | 1.00 (0.89, 1.14) |
|  | LGB | **2.48 (1.89, 3.23)** |  | 1.02 (0.85, 1.23) |
|  | Sexually diverse | **2.22 (1.54, 3.21)** |  | 0.97 (0.82, 1.16) |
| **Race and ethnicity** | |  |  |  |
|  | White | Ref |  | Ref |
|  | Black or African American | 0.70 (0.49, 1.00) |  | **0.74 (0.62, 0.89)** |
|  | Hispanic or Latino | **0.54 (0.40, 0.71)** |  | **0.77 (0.66, 0.90)** |
|  | Asian or Pacific Islander | **0.49 (0.29, 0.82)** |  | 0.84 (0.57, 1.25) |
|  | Multiracial (non-Hispanic) | 1.36 (0.92, 2.00) |  | 1.04 (0.81, 1.32) |
|  | American Indian or Alaska Native | 0.86 (0.33, 2.29)^d^ |  | 0.91 (0.61, 1.36) |
| Notes: | |  |  |  |
| Bolded outcomes indicates differences at *P*<.05 | | |  |  |
| a. The number of respondents who did not provide information about service use outcomes were as follows: digital mental health use (n=682) and digital health use (n=636). | | | | |
| b. Sexual orientation estimates adjusted for race and ethnicity, sex, age, mental health need, device or internet access, parental job loss or unemployment, English language proficiency, housing instability. | | | | |
| c. Race and ethnicity estimates adjusted for sex, age, mental health need, device or internet access, parental job loss or unemployment, English language proficiency, housing instability. | | | | |
| d. Estimate is based on the occurrence of ≤10 unweighted events and should be interpreted with caution. See Figure 1 for unweighted frequencies and weighted percentages. | | | | |
