# Supplementary material for: Intersectional Disparities in Digital Health and Mental Health Service Use Among US Youth During the COVID-19 Pandemic: Cross-Sectional Analysis of a National Survey
Source: J Med Internet Res. 2025 Oct 27;27:e77062. doi: 10.2196/77062 (PMC12603589; doi:10.2196/77062)
Supplement: Multimedia Appendix 6 [file jmir_v27i1e77062_app6.docx]

| **Multimedia Appendix 6.** Sensitivity analysis, adjusted prevalence ratios (aPRs) of digital mental health and digital health service use within racial and ethnic subgroups by sexual orientation. Cross-sectional analysis of the Adolescent Behaviors and Experiences Survey (ABES), United States, January-June 2021. | | | | |
| --- | --- | --- | --- | --- |
|  |  | **Digital mental health service use^a^** |  | **Digital health**  **service use^a^** |
|  | | aPR (95% CI)^b^ |  | aPR (95% CI)^b^ |
| **White** | |  |  |  |
|  | Heterosexual | Ref |  | Ref |
|  | All sexual minority youth | **2.62 (1.84, 3.72)** |  | 1.06 (0.88, 1.27) |
|  | LGB | **2.49 (1.68, 3.67)** |  | 1.04 (0.86, 1.36) |
|  | Sexually diverse | **2.79 (1.89, 4.14)** |  | 1.08 (0.86, 1.36) |
| **Black or African American** | |  |  |  |
|  | Heterosexual | Ref |  | Ref |
|  | All sexual minority youth | **3.22 (1.52, 6.82)** |  | 1.39 (0.93, 2.09) |
|  | LGB | **3.58 (1.61, 7.95)** |  | 1.41 (0.87, 2.28) |
|  | Sexually diverse | 2.30 (0.84, 6.28)^c^ |  | 1.35 (0.71, 2.57) |
| **Hispanic or Latino** | |  |  |  |
|  | Heterosexual | Ref |  | Ref |
|  | All sexual minority youth | 1.49 (0.78, 2.83) |  | 0.84 (0.65, 1.09) |
|  | LGB | **1.97 (1.01, 3.86)** |  | 0.93 (0.66, 1.30) |
|  | Sexually diverse | 0.83 (0.34, 2.02) |  | 0.73 (0.49, 1.04) |
| **Asian or Pacific Islander** | |  |  |  |
|  | Heterosexual | Ref |  | Ref |
|  | All sexual minority youth | 1.88 (0.41, 8.58)^c^ |  | 0.85 (0.58, 1.25) |
|  | LGB | 2.52 (0.57, 11.01)^c^ |  | 0.87 (0.61, 1.23) |
|  | Sexually diverse | 0.92 (0.12, 6.80)^c^ |  | 0.82 (0.40, 1.72)^c^ |
| **Multiracial (non-Hispanic)** | |  |  |  |
|  | Heterosexual | Ref |  | Ref |
|  | All sexual minority youth | **3.05 (1.52, 6.15)** |  | 0.74 (0.45, 1.22) |
|  | LGB | **3.38 (1.59, 7.20)** |  | 0.75 (0.38, 1.45) |
|  | Sexually diverse | 2.40 (0.93, 6.16)^c^ |  | 0.74 (0.43, 1.27) |
| **American Indian or Alaska Native** | |  |  |  |
|  | Heterosexual | Ref |  | Ref |
|  | All sexual minority youth | nr^d^ |  | nr^d^ |
|  | LGB | nr^d^ |  | nr^d^ |
|  | Sexually diverse | nr^d^ |  | nr^d^ |
| Notes: | |  |  |  |
| Bolded outcomes indicates differences at *P*<.05 | | | | |
| a. The number of respondents who did not provide information about service use outcomes were as follows: digital mental health use (n=682) and digital health use (n=636). | | | | |
| b. Estimates adjusted for sex, age, mental health need, device or internet access, parental job loss or unemployment, English language proficiency, housing instability. | | | | |
| c. Estimate is based on the occurrence of ≤10 unweighted events and should be interpreted with caution. See Figure 2 for unweighted frequencies and weighted percentages. | | | | |
| d. Estimate not reported (nr) because the unweighted denominator (n) is <30, per CDC suppression guidance for ABES. See Figure 2 for unweighted frequencies and weighted percentages. | | | | |
