# Supplementary material for: Intersectional Disparities in Digital Health and Mental Health Service Use Among US Youth During the COVID-19 Pandemic: Cross-Sectional Analysis of a National Survey
Source: J Med Internet Res. 2025 Oct 27;27:e77062. doi: 10.2196/77062 (PMC12603589; doi:10.2196/77062)
Supplement: Multimedia Appendix 7 [file jmir_v27i1e77062_app7.docx]

| **Multimedia Appendix 7.** Sensitivity analysis, adjusted prevalence ratios (aPRs) of digital mental health and digital health service use within sexual orientation subgroups by race and ethnicity. Cross-sectional analysis of the Adolescent Behaviors and Experiences Survey (ABES), United States, January-June 2021. | | | | |
| --- | --- | --- | --- | --- |
|  |  | **Digital mental health services use^a^** |  | **Digital health service use^a^** |
|  | | aPR (95% CI)^b^ |  | aPR (95% CI)^b^ |
| **Heterosexual** | |  |  |  |
|  | White | Ref |  | Ref |
|  | Black or African American | 0.90 (0.53, 1.52) |  | **0.70 (0.57, 0.86)** |
|  | Hispanic or Latino | 0.73 (0.51, 1.05) |  | **0.78 (0.65, 0.94)** |
|  | Asian or Pacific Islander | 0.78 (0.36, 1.67) |  | 0.91 (0.62, 1.33) |
|  | Multiracial (non-Hispanic) | 1.25 (0.64, 2.45) |  | 1.10 (0.84, 1.46) |
|  | American Indian or Alaska Native | 0.80 (0.33, 1.92)^c^ |  | 1.12 (0.80, 1.55) |
| **All sexual minority youth** | |  |  |  |
|  | White | Ref |  | Ref |
|  | Black or African American | 0.60 (0.35, 1.03) |  | 0.85 (0.64, 1.13) |
|  | Hispanic or Latino | **0.36 (0.23, 0.56)** |  | **0.74 (0.60, 0.91)** |
|  | Asian or Pacific Islander | **0.24 (0.07, 0.86)^c^** |  | 0.75 (0.42, 1.36) |
|  | Multiracial (non-Hispanic) | 1.25 (0.85, 1.84) |  | 0.83 (0.54, 1.25) |
|  | American Indian or Alaska Native | nr^d^ |  | nr^d^ |
| **LGB** | |  |  |  |
|  | White | Ref |  | Ref |
|  | Black or African American | 0.72 (0.37, 1.39) |  | 0.86 (0.58, 1.29) |
|  | Hispanic or Latino | **0.52 (0.31, 0.88)** |  | 0.83 (0.60, 1.17) |
|  | Asian or Pacific Islander | 0.37 (0.10, 1.40)^c^ |  | 0.78 (0.54, 1.12) |
|  | Multiracial (non-Hispanic) | 1.53 (0.89, 2.64) |  | 0.85 (0.45, 1.60) |
|  | American Indian or Alaska Native | nr^d^ |  | nr^d^ |
| **Sexually diverse** | |  |  |  |
|  | White | Ref |  | Ref |
|  | Black or African American | **0.37 (0.15, 0.90)^c^** |  | 0.86 (0.48, 1.55) |
|  | Hispanic or Latino | **0.18 (0.08, 0.40)** |  | **0.62 (0.39, 0.97)** |
|  | Asian or Pacific Islander | **0.11 (0.02, 0.63)^c^** |  | 0.75 (0.22, 2.57)^c^ |
|  | Multiracial (non-Hispanic) | 0.93 (0.48, 1.78)^c^ |  | 0.76 (0.46, 1.26) |
|  | American Indian or Alaska Native | nr^d^ |  | nr^d^ |
| Notes: | |  |  |  |
| Bolded outcomes indicates differences at *P*<.05 | | |  |  |
| a. The number of respondents who did not provide information about service use outcomes were as follows: digital mental health use (n=682), digital health use (n=636). | | | | |
| b. Estimates adjusted for sex, age, mental health need, device or internet access, parental job loss or unemployment, English language proficiency, housing instability | | | | |
| c. Estimate is based on the occurrence of ≤10 unweighted events and should be interpreted with caution. See Figure 2 for unweighted frequencies and weighted percentages. | | | | |
| d. Estimate not reported (nr) because the unweighted denominator (n) is <30, per CDC suppression guidance for ABES. See Figure 2 for unweighted frequencies and weighted percentages. | | | | |
